# Supplementary material for: No phenotypes in Ttc22 knockout mice
Source: PLoS One. 2026 Oct 23;21(10):e0361340. doi: 10.1371/journal.pone.0361340 (PMC13693868; doi:10.1371/journal.pone.0361340)

File: mouse-Ttc22-FL-(CMV-F).ab1   Run Ended: Jan 4, 2026, 6:17:44   Signal G:953 A:817 T:812 C:796
Sample: 07F_B-......419_CMV-F_TSS20260103-027-00005   Lane: 53   Base spacing: 16.60   835 bases in 12264 scans

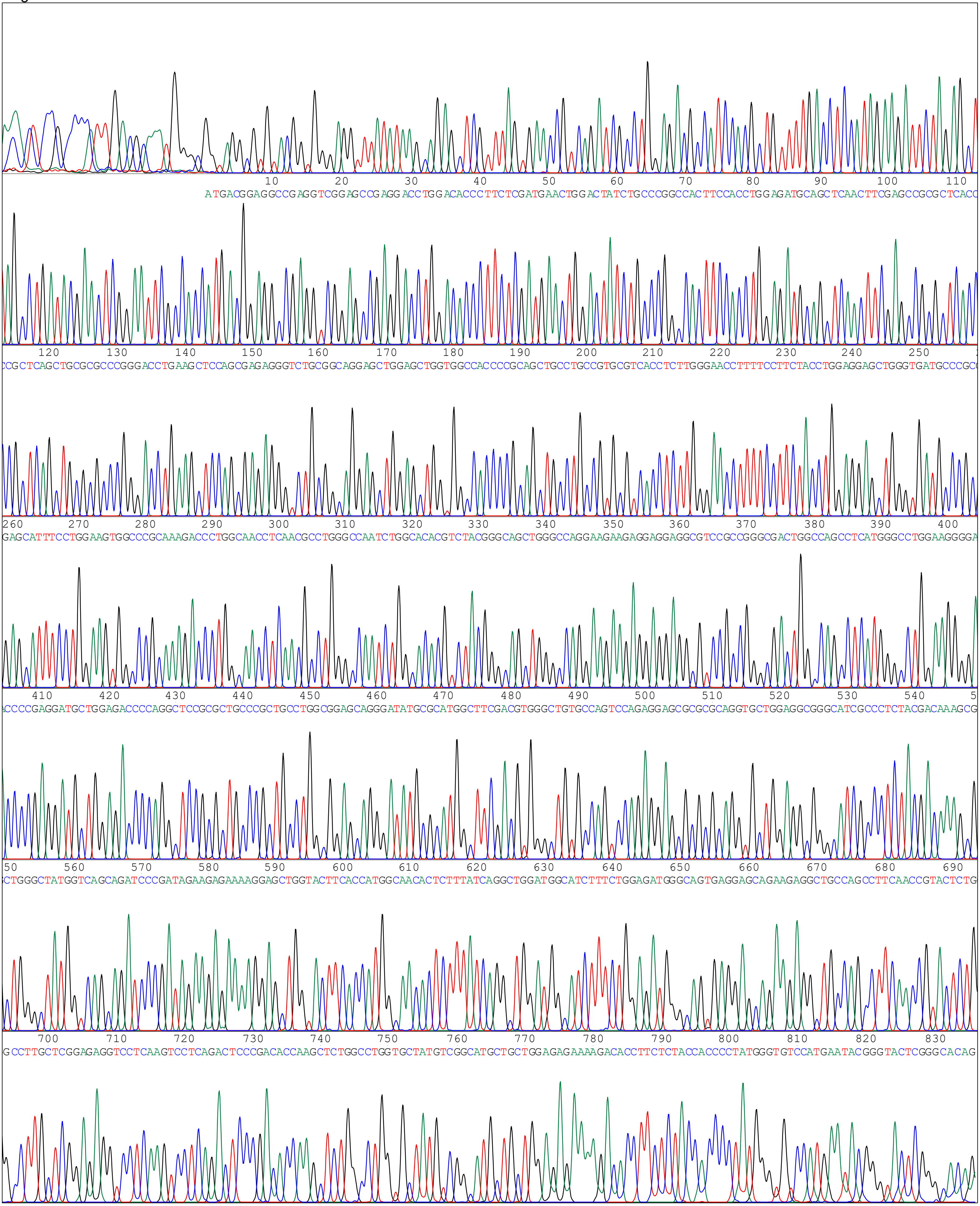

Supplement: Supplementary file 1 [file pone.0361340.s001.pdf]
