# Supplementary material for: No phenotypes in Ttc22 knockout mice
Source: PLoS One. 2026 Oct 23;21(10):e0361340. doi: 10.1371/journal.pone.0361340 (PMC13693868; doi:10.1371/journal.pone.0361340)

File: mouse-Ttc22-TPR1-(CMV-F).ab1 Run Ended: Dec 29, 2025, 5:27:52 Signal G:443 A:442 T:333 C:343
Sample: 06D_B-......291_CMV-F_TSS20251228-027-07917 Lane: 42 Base spacing: 16.39 898 bases in 12930 scans

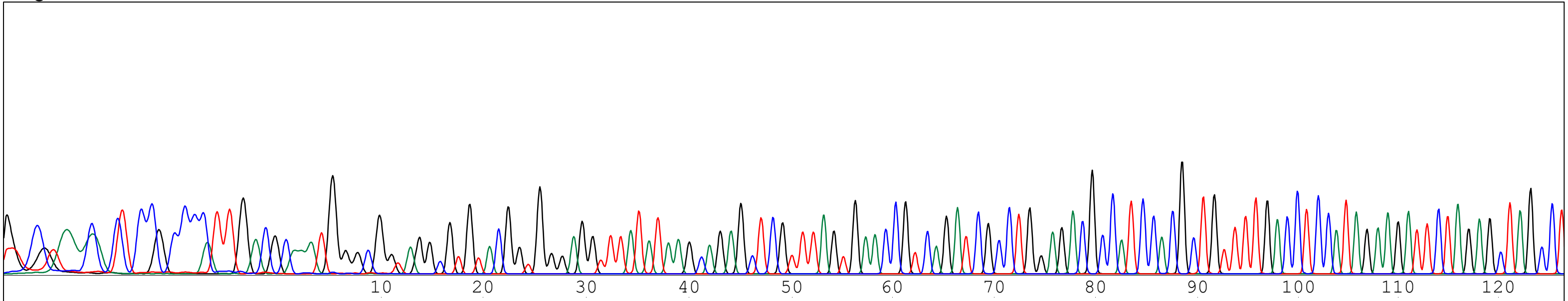

CTCAACGCCTGGGCCAATCTGGCACACGTCTACGGGCAGCTGGGCCAGGAAGAAGAGGAGGAGGCGTCCGCCGGGCGACTGGCCAGCCTCATGGGCCTGGAAGGGGACCCCGAGGATGCTGGAGAC

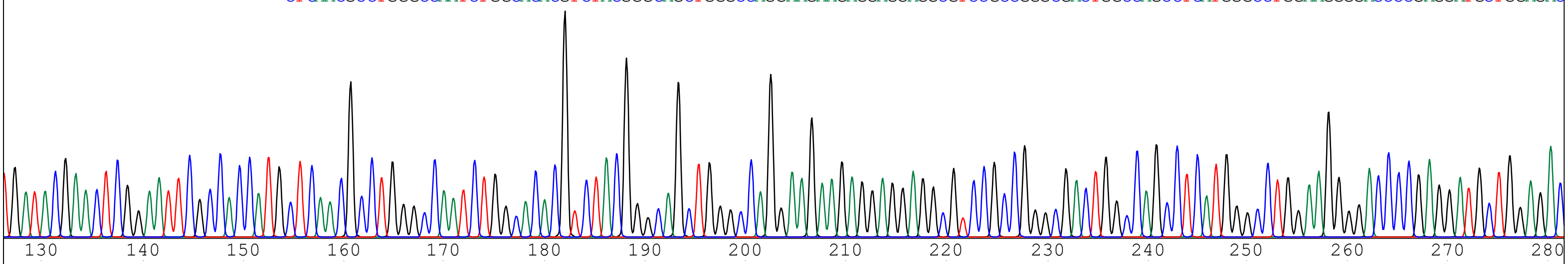

CCCCAGGCTCCGCGCTGCCCGCTGCCTGGCGGAGCAGGGATATGCGCATGGCTTCGACGTGGGCTGTGCCAGTCCAGAGGAGCGCGCGCAGGTGCTGGAGGCGGGCATCGCCCTCTACGACAAAGCGCTGGGCTATGGTCAGCAGATCCCGATAGA

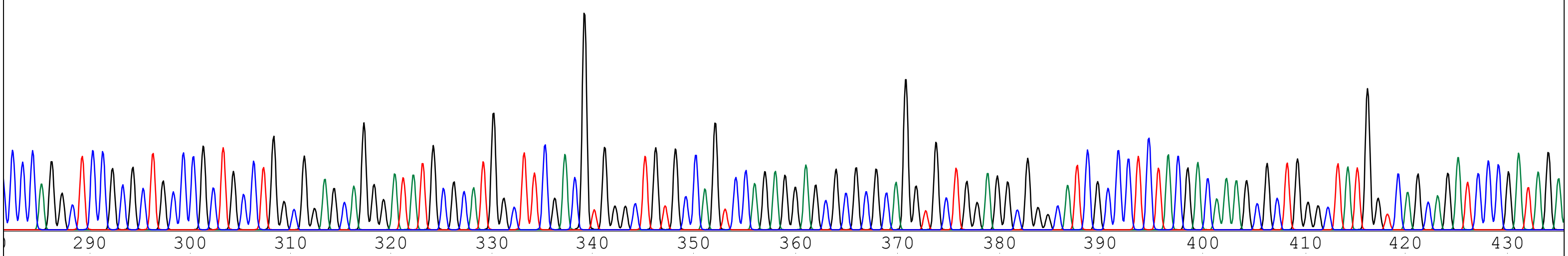

AGAGAAAAGGAGCTGGTACTTCACCATGGCAACACTCTTTATCAGGCTGGATGGCATCTTTCTGGAGATGGGCAGTGAGGAGCAGAAGAGGCTGCCAGCCTTCAACCGTACTCTGGCCTTGCTCGGAGAGGTCCTCAAGTCCTCAGACTCCCGA

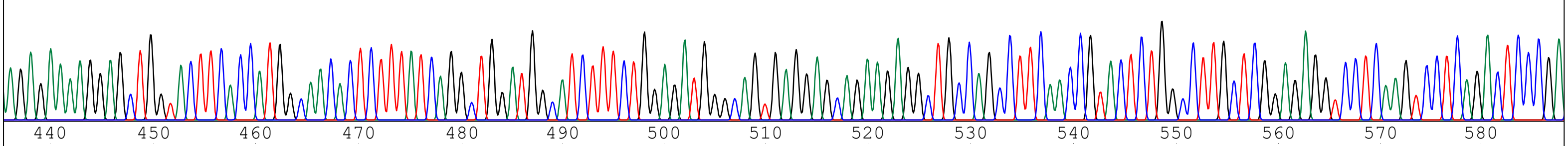

CACCAAGCTCTGGCCTGGTGCTATGTCGGCATGCTGCTGGAGAGAAAAGACACCTTCTCTACCACCCCTATGGGTGTCCATGAATACGGGTACTCGGGCACAGAGCCCCTGGACTGCTTTGGCAAGGCCATTGAGATCGCCAAGAACCAACCT

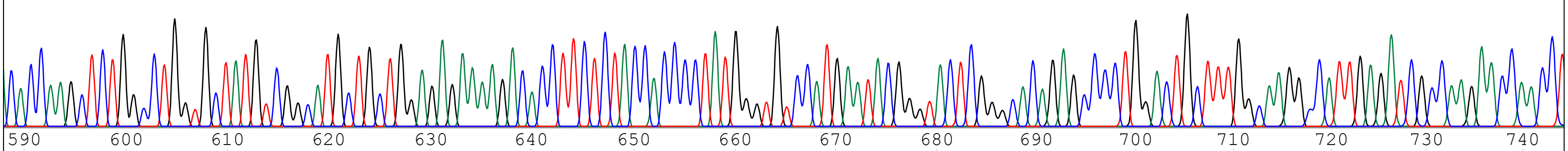

TCCCATTCTGAATCGCCTGGCCAAAATCTTCCACTTCTTGGGAAAGCAGGACATGGCCGTAGGAACCTGCAACATGGTCCTGGCGGTGCTTACAGACCCAGAGCTCAACTGGCAGGCATACTGCACCAGGGCCAAGGTCCGAATCAGAGCCTATGTC

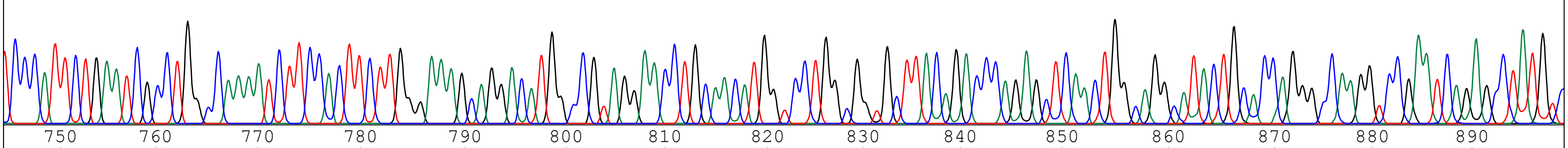

CATGACTTGGAACGAGCCAGGGGGGCTCGGGGTTTGCCTGACGGAACCCCTGGCCTGTGCCAAGCTGACCTGAGGAAGGGGCAAGGTGGGCCAAGCTCCGGACTACCAAAATCAGCAGGTTACACTACTGGGTGGGATGCCTGCGGAATTCTG

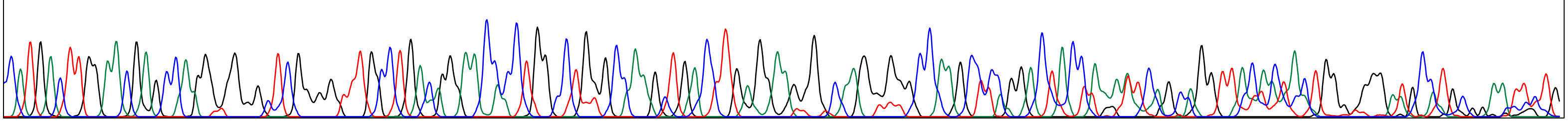

Supplement: Supplementary file 2 [file pone.0361340.s002.pdf]
