# Supplementary material for: No phenotypes in Ttc22 knockout mice
Source: PLoS One. 2026 Oct 23;21(10):e0361340. doi: 10.1371/journal.pone.0361340 (PMC13693868; doi:10.1371/journal.pone.0361340)

File: mouse-Ttc22-TPR4-(CMV-F).ab1
Sample: 06E_B-......15_CMV-F_TSS20251223-027-06342

Run Ended: Dec 24, 2025, 3:28:30
Lane: 40 Base spacing: 16.38

Signal G:1753 A:2099 T:1758 C:1772
853 bases in 12535 scans

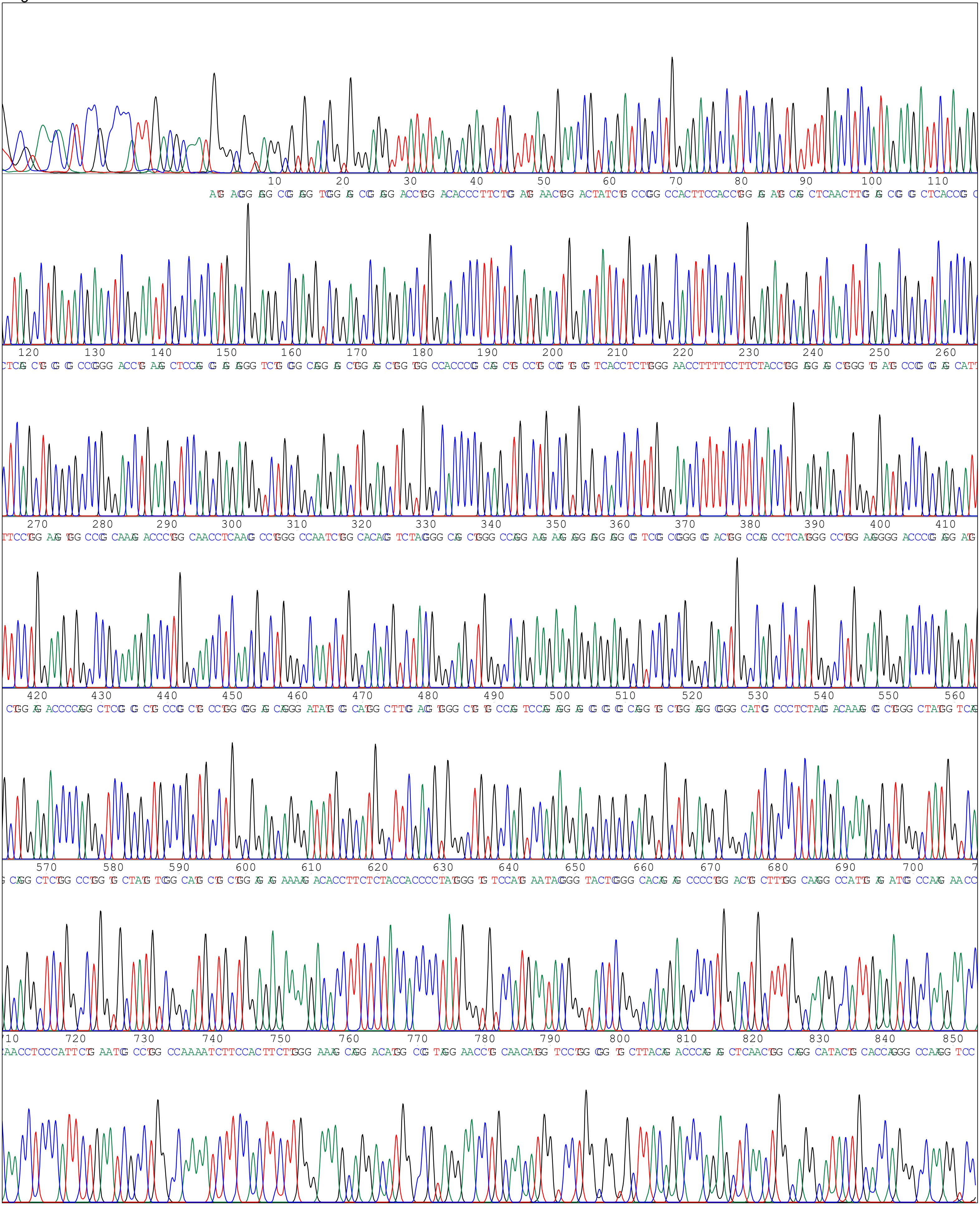

Supplement: Supplementary file 3 [file pone.0361340.s003.pdf]
